# Supplementary material for: Parental Death and Premature Mortality in Individuals with Out-of-Home Care Experience in Sweden: A Nationwide Cohort Study
Source: Int J Environ Res Public Health. 2025 Apr 7;22(4):580. doi: 10.3390/ijerph22040580 (PMC12027392; doi:10.3390/ijerph22040580)
Supplement: Supplementary file 1 [file ijerph-22-00580-s001.zip › ijerph-3486541-supplementary.pdf]

Table S1. Average follow-up, by OHC experience and sex.

	Men (n=486,841)				Women (n=461,642)			
	**Mean**	**Median**	**Min**	**Max**	**Mean**	**Median**	**Min**	**Max**
**Out-of-home care (OHC)**								
No OHC	42.04	42.29	20.00	46.96	42.13	42.29	20.02	46.96
Early short-term placement	42.33	43.00	20.20	46.96	42.63	43.21	22.65	46.96
Early intermediate placement	41.50	41.96	20.22	46.96	41.93	42.12	24.19	46.96
Long-term placement	41.78	42.29	20.04	46.96	42.02	42.46	20.07	46.96
Teenage placement	40.66	41.13	20.02	46.96	41.35	41.29	20.02	46.96

Note OHC=Out-of-home care.

Table S2. OHC and premature mortality: Moderation by parental death. Predictive margins. Results from Cox regression analysis, by sex.

	**Men (n=486,841)**			**Women (n=461,642)**		
		HR (95% CI)	HR (95% CI)		HR (95% CI)	HR (95% CI)
**Variables**	**Deaths (n)**	**Unadjusted**	**Adjusted**	**Deaths (n)**	**Unadjusted**	**Adjusted**
No OHC + no parental death (Ref)	8492	1.00	1.00	3983	1.00	1.00
No OHC + parental death	579	1.62 (1.40–1.85)	1.37 (1.13–1.60)	295	1.55 (1.22–1.88)	1.34 (1.00–1.68)
Early short-term + no parental death	126	2.46 (1.99–2.93)	1.49 (1.16–1.81)	63	2.38 (1.69–3.06)	1.59 (1.07–2.12)
Early short-term + parental death	19	3.86 (1.34–6.39)	1.98 (0.67–3.30)	10	2.56 (0.99–6.12)	1.44 (0.57–3.46)
Early intermediate + no parental death	124	4.35 (3.50–5.20)	2.31 (1.80–2.82)	37	3.26 (2.07–4.45)	1.96 (1.17–2.75)
Early intermediate + parental death	21	4.24 (0.85–7.64)	2.05 (0.40–3.70)	11	7.08 (0.87–13.30)	4.03 (0.43–7.63)
Long-term + no parental death	157	3.70 (3.04–4.35)	1.80 (1.42–2.18)	83	4.57 (3.43–5.72)	2.68 (1.86–3.50)
Long-term + parental death	25	3.22 (0.40–6.04)	1.46 (0.17–2.75)	13	2.89 (0.12–6.90)	1.63 (0.65–3.90)
Teenage + no parental death	551	**7.57** (6.89–8.26)	4.74 (4.09–5.39)	173	4.80 (4.02–5.57)	3.47 (2.70–4.24)
Teenage + parental death	76	7.78 (4.72–10.83)	4.30 (2.55–6.03)	20	3.24 (0.40–6.08)	2.32 (0.25–4.39)

Note: OHC=Out-of-home care; Ref=Reference category; HR=Hazard ratio; CI=Confidence interval. Control estimates become the same as in Table 2.
